# A Specific for Scarlet Fever and Diphtheria

**Published:** 1886-11

**Authors:** 


### *A SPECIFIC FOR SCARLET FEVER AND DIPHTHERIA.*

Dr. C. R. Illingworth, writes thus to the *Med. Press and Circular*: I find that the biniodide of mercury is a specific and prophylactic for scarlet fever and diphtheria.

Both are diseases due to the development of germs in the blood, myriads of minute nucleated bodies in active movement being visible by the microscope on examination of the membrane peculiar to each. Hence, I think, the efficacy of the remedy I name.

As all diseases of this nature deprive the blood of a large portion of its hæmoglobin and fibrin, I prescribe the ammonio-citrate of iron with it. Thus:

| | | |
|---|---|---|
| ℞ | Sol. Hydrarg. Bichlor., - - - | ʒ iij. |
| | Potass. Iodid, - - - - | gr. x. |
| | Ferri am. Citrat, - - - - | gr. xx. |
| | Syrupi, - - - - - | ℥ ss. |
| | Aquam ad, - - - - - | ℥ ij. |

et solve. fiat. mist.

Sig:—One teaspoonful every two hours (for a child of two to four years.)

As soon as all the membraneous deposit has disappeared from the parts affected I give the usual steel and chlorate of potash mixture. As a rule this occurs in from four to five days, but in severe cases it takes ten.

The only and important exception to this rule of treatment is in those cases where the disease is ushered in with vomiting and purging, with scanty rash and collapse. In these, which evidence a rapid liquefaction of the blood by the action of the poison, the iron and chlorate of potash mixture should be given at once in full doses every two hours.

